# Estimates of the genetic contribution to methane emission in dairy cows: a meta-analysis

**DOI:** 10.1038/s41598-022-16778-z

**Published:** 2022-07-19

**Authors:** Navid Ghavi Hossein-Zadeh

**Affiliations:** Department of Animal Science, Faculty of Agricultural Sciences, University of Guilan, Rasht, 41635-1314 Iran

**Keywords:** Animal breeding, Heritable quantitative trait

## Abstract

The present study aimed to perform a meta-analysis using the three-level model to integrate published estimates of genetic parameters for methane emission traits [methane yield (METY), methane intensity (METINT), and methane production (METP)] in dairy cows. Overall, 40 heritability estimates and 32 genetic correlations from 17 papers published between 2015 and 2021 were used in this study. The heritability estimates for METY, METINT, and METP were 0.244, 0.180, and 0.211, respectively. The genetic correlation estimates between METY and METINT with corrected milk yield for fat, protein, and or energy (CMY) were negative (− 0.433 and − 0.262, respectively). Also, genetic correlation estimates between METINT with milk fat and protein percentages were 0.254 and 0.334, respectively. Although the genetic correlation estimate of METP with daily milk yield was 0.172, its genetic correlation with CMY was 0.446. All genetic correlation estimates between METP with milk fat and protein yield or percentage ranged from 0.005 (between METP-milk protein yield) to 0.185 (between METP-milk protein percentage). The current meta-analysis confirmed the presence of additive genetic variation for methane emission traits in dairy cows that could be exploited in genetic selection plans.

## Introduction

In agriculture, dairy cattle are responsible for a significant portion of universal greenhouse gas emissions as enteric methane^1^. Therefore, there has been a greater focus on the dairy industry to improve the efficiency of production^2^. When human inedible plant materials are converted into energy by cattle, six to 11% of the feed energy is lost to methane emissions^3^. If methane production can be reduced, the retained energy of feed held by the animal could be utilized for milk production, growth, etc.^4,5^.

During the last two decades, the breeding objectives of dairy cows have changed substantially and included many traits such as health, fertility, and longevity^6^. These changes are necessary to provide greater profit for farmers and increase animal welfare. In addition, based on the demands of the dairy industry and relevant technical improvements, selection indices of dairy cattle are modified. The focus of new breeding goals in dairy cattle is on profitability improvement, environmental effects, animal welfare, and health^6^. However, breeding objectives for environmental traits are in their early stages. Genetic gains in production and reproduction performance and health traits of dairy cows have indirectly decreased the environmental impact of dairy cattle^1^. Therefore, the main objective of the dairy enterprise is to enhance the total profitability and stability of dairy production by reducing methane emissions without any negative influence on economically important traits. Reaching this goal is possible by including a methane emission trait in the selection indices^7^. Studies have suggested including traits related to the environment, chiefly methane production, into breeding objectives^8–10^. Also, several studies suggested genetic variation for methane traits in dairy cattle^11–13^. Therefore, methane emission could be considered an appropriate candidate trait to decline via genetic selection.

A meta-analysis consists of a multi-step process and a group of statistical methods for integrating the results of separate studies to reach general conclusions on a particular subject. Different steps of a meta-analysis can be considered as follows: (1) designing a research question; (2) trying to find proper studies; (3) extracting required information from the studies; (4) combining the obtained information in the analysis models; and (5) explaining the outputs from the meta-analysis and making general conclusions^14^.

Accurate estimates of genetic parameters for traits of economic importance are essential in genetic selection schemes. There are several reports of genetic parameter estimates for methane-related traits in different dairy cow populations. Nevertheless, these estimates have been obtained from studies containing data from cows of different breeds and lactation numbers, with a different number of observations and with diverse variables in the analysis model. This variability among the studies has led to considerable variation among the heritability estimates and genetic correlations. The logic for planning the present study was the requirement for integrating estimates from former studies to prepare summary genetic parameter estimates for methane emission traits to help establish breeding goals for dairy cattle. In addition, to prevent inappropriate correlated responses with other economically important traits, recognizing the genetic associations with production traits is essential before implementing methane into the breeding objectives of dairy cows. The present study aimed to perform a meta-analysis using the three-level model to integrate published estimates of genetic parameters for methane emission traits in dairy cows.

## Materials and methods

### Characterizing the scope of the meta-analysis study

**Figure 1:**
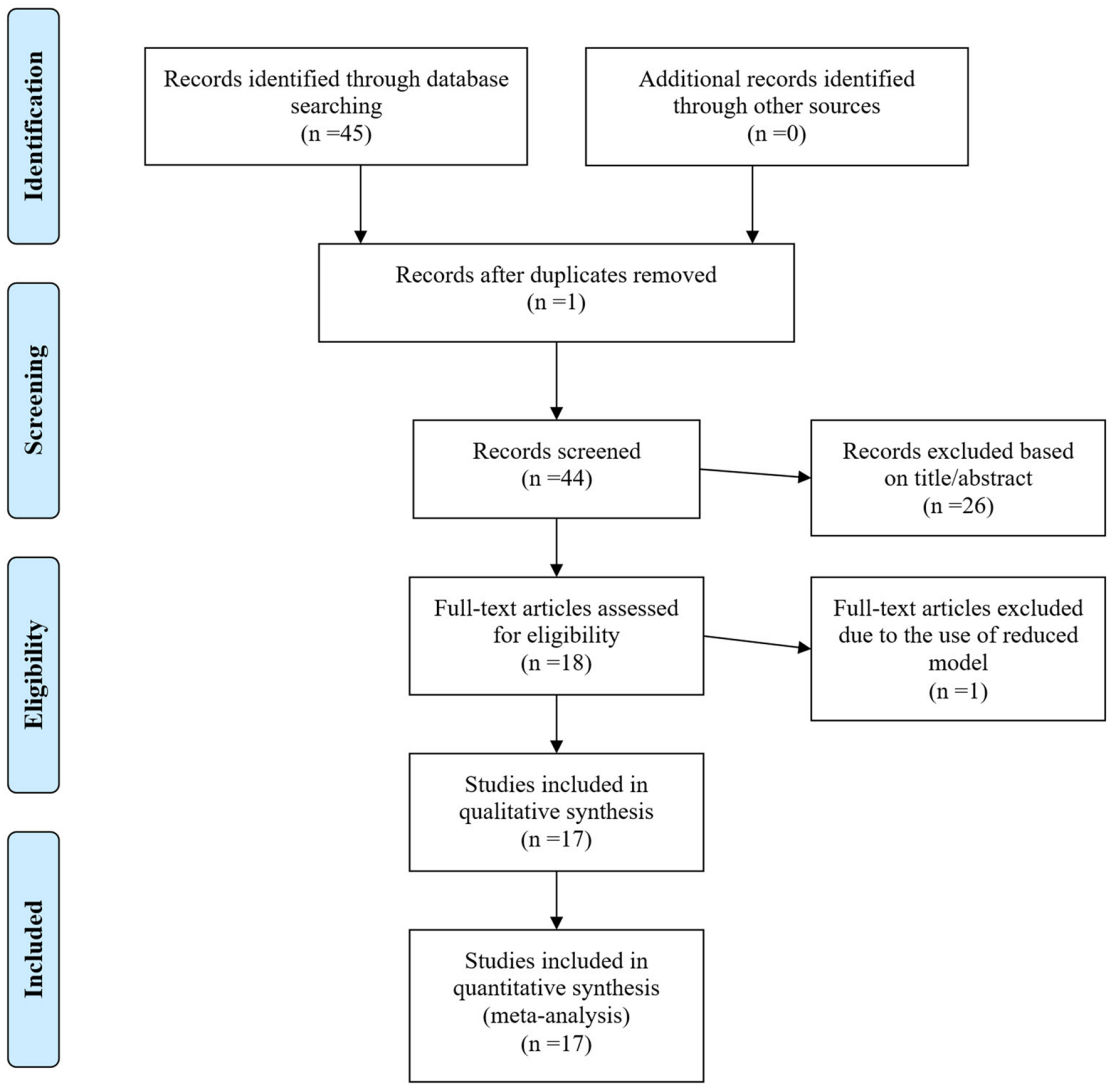
The PRISMA flowchart to describe the process of study selection and systematic literature search.

The PRISMA (Preferred Reporting Items for Systematic Reviews and Meta-Analyses) guideline to address meta-analyses and systematic reviews was used in this study (Fig. 1) ^15^. A systematic literature search using electronic databases of ISI Web of Knowledge (https://apps.webofknowledge.com), Google Scholar (https://scholar.google.com), and NCBI (https://www.ncbi.nlm.nih.gov) was performed to recognize all citations reporting heritability estimates for methane emission traits and their genetic association with milk yield and composition of dairy cows. A comprehensive search was conducted with the following keywords and their synonyms or derivatives: “dairy cow”, “methane emission”, “enteric methane”, “methane production”, “methane intensity”, “methane yield”, “genetic parameters”, “heritability”, and “genetic correlation”. Overall, 40 heritability estimates and 32 genetic correlations from 17 papers were used in this meta-analysis study. The included papers were published between 2015 and 2021. The characteristics of studies included in the database for conducting this meta-analysis are indicated in Table 1. The genetic parameter estimates were obtained from mixed animal models based on the Bayesian inference and restricted maximum likelihood (REML) estimation methodologies. Thus, parameter estimates from reduced models were excluded. Only papers published in scientific index journals were used, and papers published in other sources were removed. The references in the above papers were also controlled. Methane emission traits considered in this study were methane yield per kg dry matter intake (METY, in g/kg), methane intensity per kg fat and protein corrected milk produced (METINT, in g/kg), and methane production as daily methane production per cow (METP, in g/day).

**Table 1:** The characteristics of studies included in the database for conducting this meta-analysis.

Reference	Traits	Details	Effect size	Study	Country	Breed	Analysis method
Bittante and Cecchinato^16^	METY, METINT, METP		1	1	Italy	Brown Swiss	Bayesian
Bittante et al.^17^	METY, METINT, METP	Data set 1	2	2	Italy	Brown Swiss	Bayesian
Bittante et al.^17^		Data set 2	3	2	Italy	Brown Swiss	Bayesian
Bittante et al.^17^		Data set 3	4	2	Italy	Brown Swiss	Bayesian
Bittante et al.^17^		Data set 4	5	2	Italy	Brown Swiss	Bayesian
Bittante et al.^17^		Data set 5	6	2	Italy	Brown Swiss	Bayesian
Bittante et al.^17^		Data set 6	7	2	Italy	Brown Swiss	Bayesian
Difford et al.^18^	METP		8	3	Denmark	Holstein	REML
Kandel et al.^19^	METINT, METP	Data set 1	9	4	Belgium	Holstein	Bayesian
Kandel et al.^19^		Data set 2	10	4	Belgium	Holstein	Bayesian
Lassen and Løvendahl^13^	METINT, METP		11	5	Denmark	Holstein	REML
Lassen et al.^20^	METINT, METP		12	6	Denmark	Holstein	REML
López-Paredes et al.^11^	METP		13	7	Spain	Holstein	REML
Manzanilla-Pech et al.^21^	METY, METINT, METP		14	8	Australia	Holstein	REML
Pickering et al.^12^	METP		15	9	United Kingdom	Holstein	REML
Pszczola et al.^22^	METP		16	10	Poland	Holstein	REML
Pszczola et al.^23^	METP		17	11	Poland	Holstein	REML
Richardson et al.^24^	METY, METINT, METP		18	12	Australia	Holstein	REML
Sypniewski et al.^25^	METP		19	13	Poland	Holstein	REML
van Engelen et al.^26^	METY	Data set 1	20	14	Netherlands	Holstein	REML
van Engelen et al.^26^		Data set 2	21	14	Netherlands	Holstein	REML
van Engelen et al.^26^		Data set 3	22	14	Netherlands	Holstein	REML
Vanrobays et al.^27^	METP		23	15	Belgium	Holstein	REML
Yin et al.^28^	METP		24	16	Switzerland	Brown Swiss	REML
Zetouni et al.^29^	METP		25	17	Denmark	Holstein	REML

METY, Methane yield; METINT, Methane intensity; METP, Methane production.

### Data recorded

The data sets included information on the estimates of heritability for METY, METINT, and METP, and also genetic associations of methane emission traits with production traits [daily milk yield (DMY), corrected milk yield for fat, protein, and or energy (CMY), milk fat percentage (Fatp), milk protein percentage (Prop), milk fat yield (Faty), and milk protein yield (Proy)], and their standard errors. Other details registered were the year of publication, journal name, the number of observations or records, phenotypic mean and standard deviation, breed name, country of origin, parity number, years of data collection, univariate or multivariate analysis model, and the estimation method. When the same estimate was reported in different publications, based on the same database, only the most recent publication was included in the analysis. The analysis was performed exclusively for traits in which the parameter estimates were placed on not less than two distinct data sets.

For articles in which the standard errors for the heritability or correlation estimates were not reported, approximated standard errors were derived by using the combined-variance method^30^, which is given by the following formula:

$$SE_{ij} = \sqrt {\frac{{\left( {\frac{{\sum\limits_{k = 1}^{K} {s_{ik}^{2} n_{ik}^{2} } }}{{\sum\limits_{k = 1}^{K} {n_{ik} } }}} \right)}}{{n^{\prime}_{ij} }}}$$

where SE_ij_ is the predicted standard error for the published parameter estimate for the *i*th trait in the *j*th article that has not reported the standard error, *s*_ik_ is the published standard error for the parameter estimate for the *i*th trait in the *k*th article that has reported the standard error, *n*_ik_ is the number of used records to predict the published parameter estimate for the *i*th trait in the *k*th article that has reported the standard error, and *n´*_ij_ is the number of used records to predict the published parameter estimate for the *i*th trait in the *j*th article that has not reported the standard error.

### Meta-analysis of heritability estimates and genetic correlations

The database used in the current study has a hierarchical structure because some effect sizes were extracted from studies conducted by the same authors (Table 1). Indeed, the true underlying effects are expected to be more similar for such studies (i.e., effect sizes are likely correlated). A three-level meta-analysis model accounted for this dependency among studies^31^. The random effects of the study and effect size were specified as a list of one-sided formulas in the random argument of the *rma.mv* function of the metafor package version 3.0-2^32^ in R software. The REML method was used and the effect sizes with the same level within each grouping variable received the same random effect; otherwise, effect sizes were assumed to be independent.

Forest plots were built to demonstrate the effect size for each study. Effect sizes in forest plots were the average estimates of heritability for methane emission traits or their genetic association with milk production traits with a 95% confidence interval.

Because of the non-normal nature of correlation estimates, almost all meta-analyses do not apply the published correlation estimates. Preferably, the reported correlation estimate is transformed to the Fisher’s *Z* scale, and every analysis is conducted with the converted values^33^. Estimated parameters and their confidence intervals would then be transformed into correlations. The Fisher’s *Z* transformation to obtain an approximate normal scale is represented as follows^33^:

$$Z_{ij} = 0.5\left[ {\ln \left( {1 + r_{{g_{ij} }} } \right) - \ln \left( {1 - r_{{g_{ij} }} } \right)} \right]$$

where r_gij_ = the reported estimate of genetic correlation for the *i*th trait in the *j*th paper. The following formula was utilized to return to the original scale:

$$r_{{g_{ij} }}^{*} = \frac{{e^{{2Z_{ij} }} - 1}}{{e^{{2Z_{ij} }} + 1}}$$

where $r_{{g_{ij} }}^{*}$ = the re-transformed genetic correlation for the *i*th trait in the *j*th article and *Z*_ij_ = the Fisher’s *Z* transformation.

The 95% lower and upper limits for the estimated parameter would be calculated respectively for each trait as follows:

$$LL_{{\overline{\theta }}} = \overline{\theta } - 1.96 \times SE_{{\overline{\theta }}} \;\;{\text{and}}\;\;UL_{{\overline{\theta }}} = \overline{\theta } + 1.96 \times SE_{{\overline{\theta }}}$$

where $SE_{{\overline{\theta }}}$ = the predicted standard error for the estimated parameter $\overline{\theta }$, given by:

$$SE_{{\overline{\theta }}} = \sqrt {\frac{1}{{\sum\limits_{j = 1}^{k} {w_{j} } }}}$$

### Heterogeneity

The I^2^ statistic was utilized to assess heterogeneity as follows^34^:

$$I^{2} \left( \% \right) = \frac{{Q - \left( {k - 1} \right)}}{Q} \times 100$$

where Q = the χ^2^ heterogeneity statistic and k is the number of studies. Q is the Q statistics given by the following formula:

$$Q = \sum\limits_{j = 1}^{k} {w_{j} \left( {\hat{\theta }_{j} - \overline{\theta }} \right)}^{2}$$

where *w*_j_ = the parameter estimate weight [assumed as the inverse of published sampling variance for the parameter $\left( {\frac{1}{{s_{j}^{2} }}} \right)$] in the *j*th article; $\hat{\theta }_{j}$ = the published estimate of the genetic parameter in the *j*th paper, $\overline{\theta }$ = the weighted mean of the parameter in the population,, and k is the number of used papers. The I^2^ statistic characterizes the variation among the studies because of heterogeneity. Negative values of I^2^ are considered zero; therefore, I^2^ values vary between 0 and 100%^35^. If the values of I^2^ fall within the range of 0–40%, there is no concern about the heterogeneity. However, the I^2^ values of 40–60% and 60–100% often represent moderate and substantial heterogeneities, respectively.

### Publication bias

Egger’s linear regression asymmetry was utilized to test the existence of publication bias. Also, to demonstrate asymmetry, funnel plots were applied. When no publication bias exists, the funnel plot presents the symmetric distribution of effect sizes around the actual effect size.

## Results

### Descriptive statistics

The number of literature estimates, measurement units, the total number of records, weighted mean, standard deviation, and the coefficient of variation for methane emission traits and milk yield and composition of dairy cows are indicated in Table 2. The weighted coefficients of variation for methane emission traits were generally low to moderate and varied from 4.60 (for METINT) to 23.64% (for METP). In addition, the weighted coefficient of variation for milk yield and composition was low and varied from 7.14 (for FY) to 15.47% (for CMY).

**Table 2:** Number of literature estimates (N), measurement units (Unit), the total number of records (Records), weighted mean, standard deviation (SD), and the coefficient of variation (CV) for methane emission traits, milk yield, and composition of dairy cows.

Trait	Unit	N	Records	Mean	SD	CV (%)
METY	g/kg	9	11,516	21.21	1.39	6.55
METINT	g/kg	10	606,144	19.34	0.89	4.60
METP	g/day	21	1,170,345	391.03	92.43	23.64
CMY	kg/day	3	4591	33.09	5.12	15.47
DMY	kg/day	5	1,745,435	27.21	2.06	7.57
FP	%	3	2931	3.81	0.36	9.45
PP	%	3	2931	3.39	0.25	7.37
FY	kg/d	3	599,679	0.98	0.07	7.14
PY	kg/d	3	599,679	0.83	0.06	7.23

METY, Methane yield; METINT, Methane intensity; METP, Methane production; CMY, Corrected milk yield for fat, protein, and or energy; DMY, Daily milk yield; FP, Fat percentage of milk; PP, Protein percentage of milk; FY, Milk fat yield; PY, Milk protein yield.

### Heritability estimates

**Figure 2:**
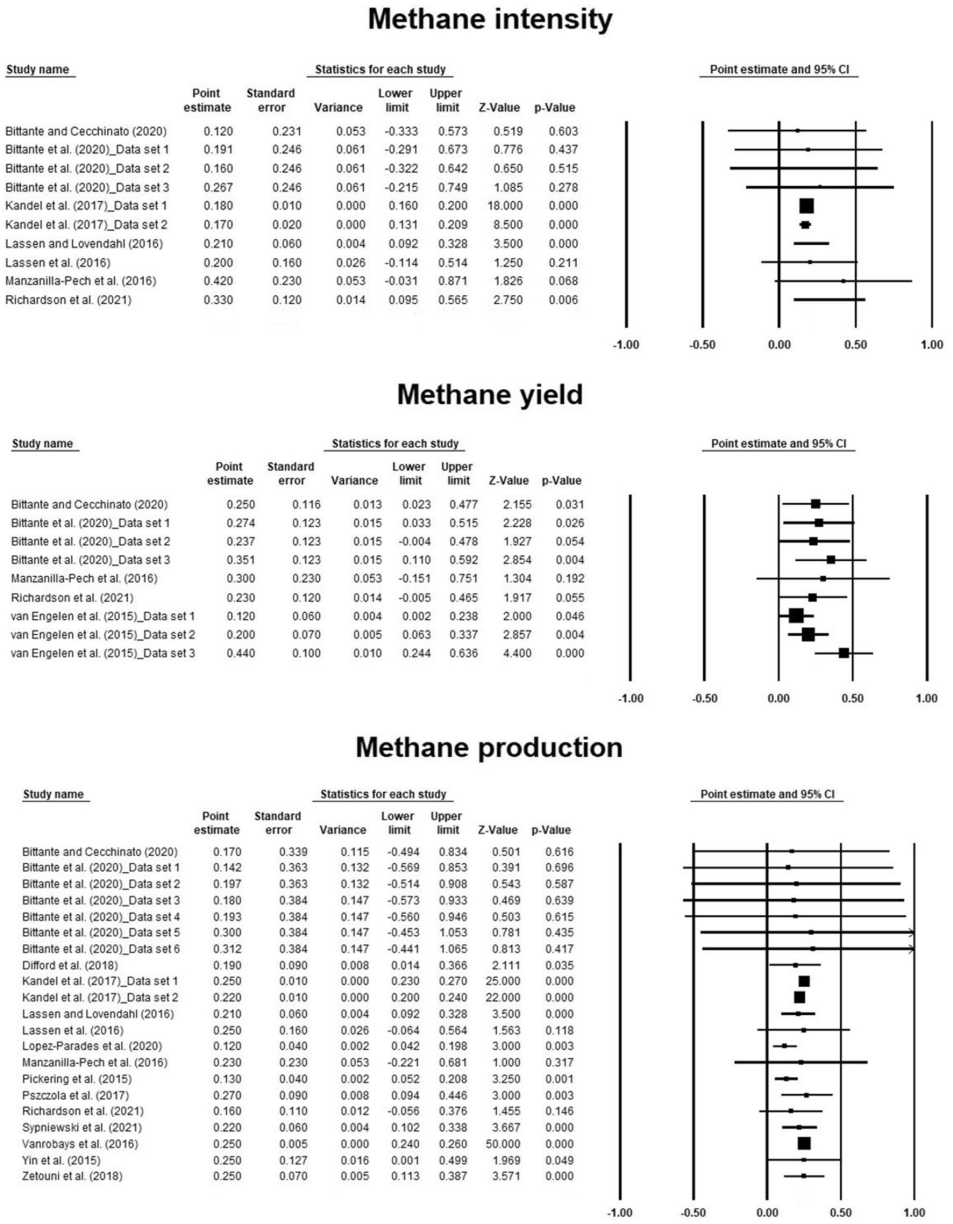
The forest plots of individual studies for the heritability estimates of METY, METINT, and METP. The weight for each study regarding the average effect size represents by the squares’ size. Bigger squares present more weight. The 95% confidence interval for each study indicates by the horizontal bars.

Effect size and heterogeneity of the heritability estimates for methane emission traits obtained from the three-level model of the meta-analysis are presented in Table 3. The heritability estimates for METY, METINT, and METP were 0.244, 0.180, and 0.211, respectively. These estimates had low standard errors, and their 95% confidence intervals were small. Also, the heritability estimates for methane emission traits were significant (*P* < 0.05). The I^2^ values showed minor heterogeneity for the heritability estimates of methane emission traits (Table 3). The results of Egger’s test for the occurrence of possible publication bias showed significant publication bias for METY (*P* = 0.088) and METP (*P* = 0.081), but non-significant publication bias (*P* = 0.125) was observed for METINT. The forest plots of individual studies for heritability estimates of METY, METINT, and METP in dairy cows are indicated in Fig. 2. Also, funnel plots of heritability estimates for METP and METINT are shown in Supplementary Figures S1 and S2, respectively.

**Table 3:** Effect size and heterogeneity of the heritability estimates for methane emission traits of dairy cows obtained from three-level model of meta-analysis.

Trait*	N	h^2^	SE	95% CI	*P*-value	I^2^
METY	9	0.244	0.041	0.150–0.339	0.000	12.711
METINT	10	0.180	0.009	0.160–0.200	0.000	0.000
METP	21	0.211	0.018	0.173–0.249	0.000	25.487

*For traits, see Table 2.

### Genetic correlation estimates

**Figure 3:**
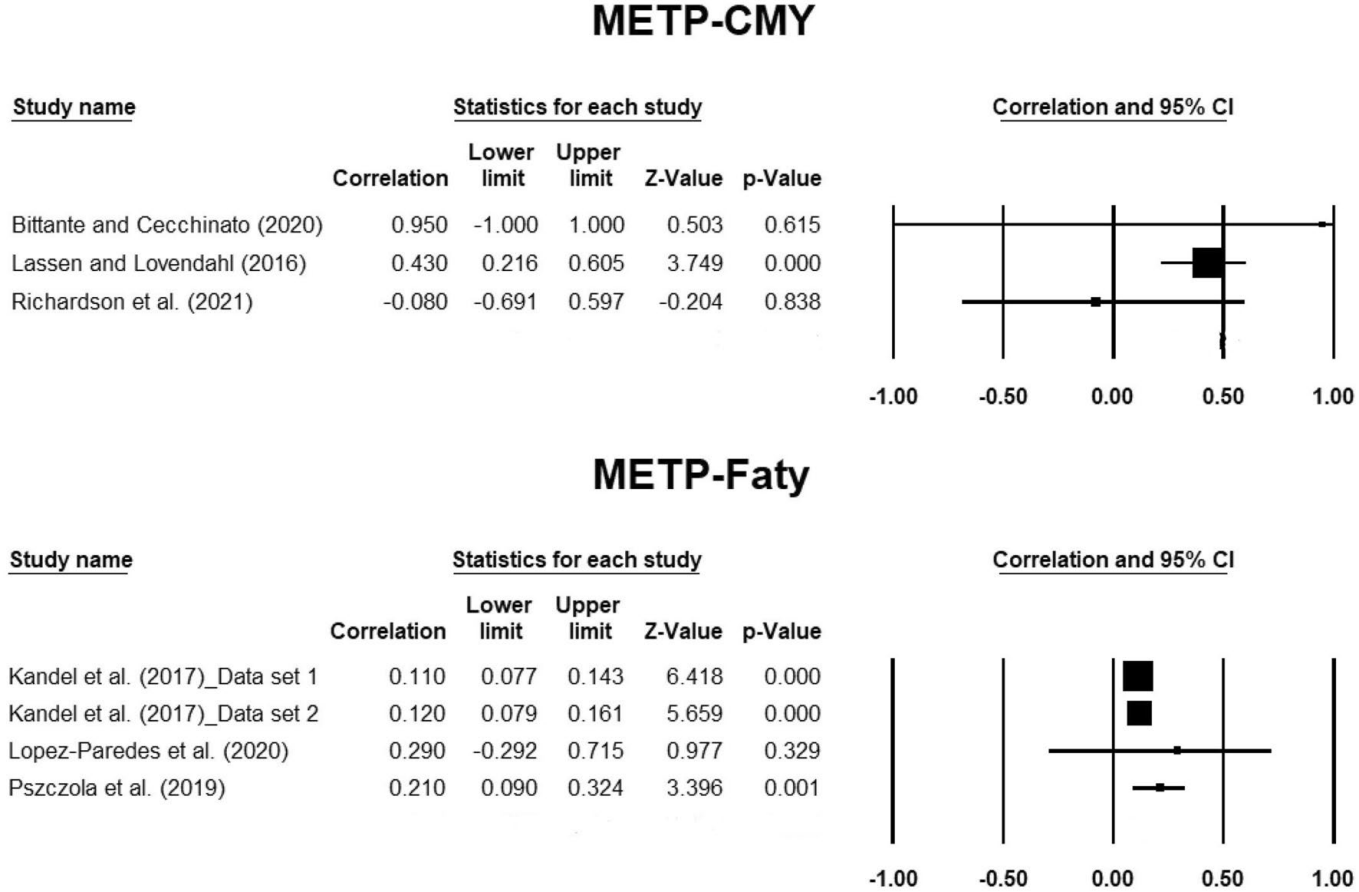
The forest plots of individual studies for the genetic correlation estimates between METP with CMY and Faty in dairy cows. Details are provided in Fig. 2.

Effect size and heterogeneity of the genetic correlation estimates between methane emission traits with milk yield and composition in dairy cows obtained from the three-level model of the meta-analysis are shown in Table 4. The genetic correlation estimates between METY-CMY and METINT-CMY were negative (− 0.433 and − 0.262, respectively). Genetic correlation estimates between METINT with Fatp and Prop were positive and moderate (Table 4). Although the genetic correlation estimate of METP with DMY was 0.172, its genetic correlation with CMY was 0.446. All genetic correlation estimates between METP with milk fat and protein yield or percentage ranged from 0.005 (between METP-milk protein yield) to 0.185 (between METP-milk protein percentage) (Table 4). Except for genetic correlations between METP-CMY and METP-Faty, other genetic correlations were non-significant (*P* > 0.05). Hence, the 95% confidence interval of non-significant genetic correlation estimates included zero. The I^2^ values indicated substantial heterogeneities for the genetic correlations between METINT-CMY and METP-DMY (Table 4). In addition, high heterogeneity was observed for the genetic correlation estimate between METINT-Prop. Also, moderate heterogeneity was observed for the genetic correlation between METP-CMY. Other genetic correlation estimates had negligible heterogeneities (Table 4). The forest plots of individual studies for genetic correlation estimates between METP-CMY and METP-Faty are depicted in Fig. 3. Also, the forest plots of individual studies for other genetic correlation estimates are displayed in Supplementary Figures S3 to S12. The results of Egger’s test showed significant (*P* = 0.000) publication bias for the genetic correlation between METP and Fatp, but non-significant (*P* > 0.10) publication bias was observed for other genetic correlation estimates.

**Table 4:** Effect size and heterogeneity of the genetic correlation estimates between methane emission traits with production traits in dairy cows obtained from the three-level model of meta-analysis.

Trait 1*	Trait 2*	N	r_g_	95% CI	*P*-value	I^2^
METY	CMY	2	− 0.433	− 0.938 to 0.073	0.093	0.000
METINT	Fatp	2	0.254	− 0.232 to 0.741	0.306	0.000
METINT	Prop	2	0.334	− 0.456 to 1.000	0.407	80.802
METINT	CMY	3	− 0.262	− 0.768 to 0.244	0.311	90.270
METP	Fatp	4	0.056	− 0.058 to 0.170	0.333	0.000
METP	Prop	4	0.185	− 0.153 to 0.522	0.283	34.783
METP	DMY	6	0.172	− 0.310 to 0.654	0.402	78.628
METP	CMY	3	0.446	0.071 to 0.820	0.020	48.000
METP	Faty	4	0.148	0.060 to 0.236	0.001	0.000
METP	Proy	4	0.005	− 0.107 to 0.116	0.934	31.772

*For traits, see Table 2. r_g_: Genetic correlation.

## Discussion

This study is the first comprehensive meta-analysis of the heritabilities for methane emission traits and their genetic correlation with milk production traits in dairy cows. Only three methane emission traits of METY, METINT, and METP were considered in this study, and residual methane production was not considered because the number of genetic parameter estimates reported for this trait was lower than the required meta-analysis standards.

Knowledge of heritability and genetic correlations with other traits of economic importance is essential to include methane emission traits in breeding goals^36^. Present breeding goals for dairy cattle do not incorporate enteric methane traits. However, the genetic improvement of farm animals is especially a constructive methodology, providing cumulative and constant modifications in the traits of interest^36^. Therefore, determining the genetic variability of methane emission traits would be the first step in including these environment-related traits in the proposed selection indices^19^. In this context, three preconditions are required. First, methane emission traits must be adequately heritable to permit a somewhat quick and meaningful improvement. Second, enough genetic variation for these traits must be proven in the studied dairy cow population. Third, genetic associations of methane emission traits with other traits of interest must be understood. The genetic analysis must confirm these three preconditions^19^. Only the relationships with milk production traits were considered in this paper.

The generally low weighted coefficients of variation for the studied traits indicated the lower dispersion around the weighted phenotypic means. This result implied the weighted phenotypic means for these traits were accurate. The low weighted coefficients of variation for METINT and METY showed that the phenotypic variation for these traits is restricted biologically. However, the highest weighted coefficient of variation was observed for METP. This result indicates that higher phenotypic variation existed in this trait than in other traits.

The current meta-analysis confirmed the presence of a genetic component for methane emission traits in dairy cows. The low heritability estimate for METINT indicated the minor influence of additive genetic effects on this trait. However, the average heritability estimates for METY and METP showed a medium effect of additive genes on these traits and a possibly suitable response to selection for them. Numerous variables may affect methane production, including measurement time, herd, diet composition, season, stage of lactation, and more. Therefore, it is necessary to adjust the relevant models of analysis for these effects when daily or lactational methane production is predicted. This adjustment will prevent bias in the phenotypic records, genetic predictions, and estimation of variance components^37^. Because meta-analysis combines published genetic parameter estimates reported by different studies, it is anticipated that the actual parameter could differ among the studies.

In general, most genetic correlation estimates had a wide 95% confidence interval which included zero. Therefore, these correlations must be interpreted with caution. The positive genetic correlation between METP and CMY likely reflects the association between methane production, energy intake, and milk yield^13^. This result would be expected because the increase in the genetic potential of animals to produce more milk increases methane emissions per animal because of an increase in feed consumption^38^. The positive genetic correlations between METP with CMY and Faty indicate that genetic and physiological mechanisms controlling these traits could be similar. It was reported that genes responsible for methane production also control lipid synthesis^39^. Milk composition and fatty acids are often used to predict methane production^40–42^. The positive genetic correlation suggests that breeding for increased milk production with higher fat content can increase methane production. The negative genetic correlations of METY and METINT with CMY indicated the opposite direction of changes when genetic selection is directly performed on milk production. Because of the negative genetic correlation between METINT-CMY, the positive genetic correlations of METINT with Fatp and Prop would be expected. The possible reasons for these positive genetic correlations would be the negative correlation between milk yield with Fatp and Protp and the dilution effect. It seems that selection for higher milk yield, and accordingly higher feed consumption and likely live weight, results in a rise in daily methane production per cow. If METP is included with a negative weight within a selection index, it would be possible to expect a decrease in the genetic gain in milk yield due to the unfavorable positive genetic correlation between these two traits.

Defining methane emission traits as ratios is a helpful metric for characterizing groups of animals, such as different treatment groups, herds, breeds, and species. However, ratio traits usually violate two statistical assumptions, which can affect the definition of the linear relationship (regression or correlation) between the two sets of traits, making them unsuitable for incorporation in genetic selection programs^43,44^. First, it is assumed that a ratio is independent, or uncorrelated, to its numerator or denominator. Second, it is assumed that the relationship between a ratio and its component traits is linear. Therefore, applying methane ratio traits in animal breeding and genetics is challenging because the complicated statistical characteristics of these ratio traits may cause unfavorable correlations (i.e., METINT may be unfavorably associated with energy-corrected milk, which is the denominator of the METINT ratio)^37^.

The meta-analysis of some genetic parameter estimates showed significant publication bias and or asymmetry in the funnel plots. Asymmetrical funnel plots may indicate publication bias or be due to exaggeration of treatment effects in small studies of low quality. Egger et al.^45^ stated that the potential sources of asymmetry in funnel plots are generally grouped as follows: 1. Selection biases, 2. True heterogeneity (effect size differs according to study size), 3. Data irregularities, 4. Artefact (heterogeneity due to poor choice of effect measure), and 5. Chance.

## Conclusion

The current meta-analysis confirmed the presence of a genetic component for methane emission traits in dairy cows that could be exploited in genetic selection plans. The positive genetic correlations between METP with CMY and Faty mean that a decrease in methane production should have adverse effects on milk and fat yields. Therefore, cows producing higher milk and milk fat are expected to emit more methane. The positive genetic correlations between METP with CMY and Faty indicate that genetic and physiological mechanisms controlling these traits could be similar.

## Supplementary Information

Supplementary Information 1.

Supplementary Information 2.

## Data Availability

All data generated or analysed during this study are included in this published article (and its Supplementary Information files).
