# Supplementary material for: Estimates of the genetic contribution to methane emission in dairy cows: a meta-analysis
Source: Sci Rep. 2022 Jul 19;12:12352. doi: 10.1038/s41598-022-16778-z (PMC9296463; doi:10.1038/s41598-022-16778-z)
Supplement: Supplementary file 1 — Supplementary Information 1. [file 41598_2022_16778_MOESM1_ESM.docx]

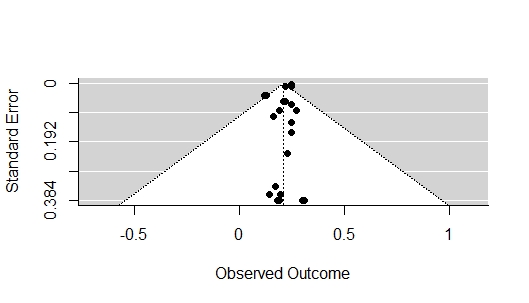


**Figure S1.** The funnel plot of the heritability estimates for METP.


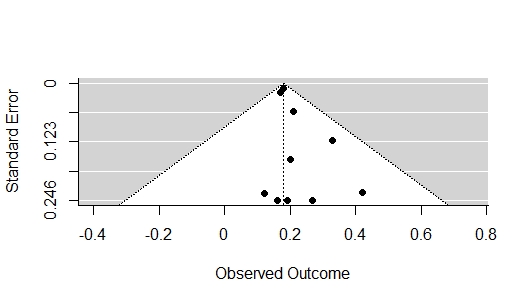


**Figure S2.** The funnel plot of the heritability estimates for METP.


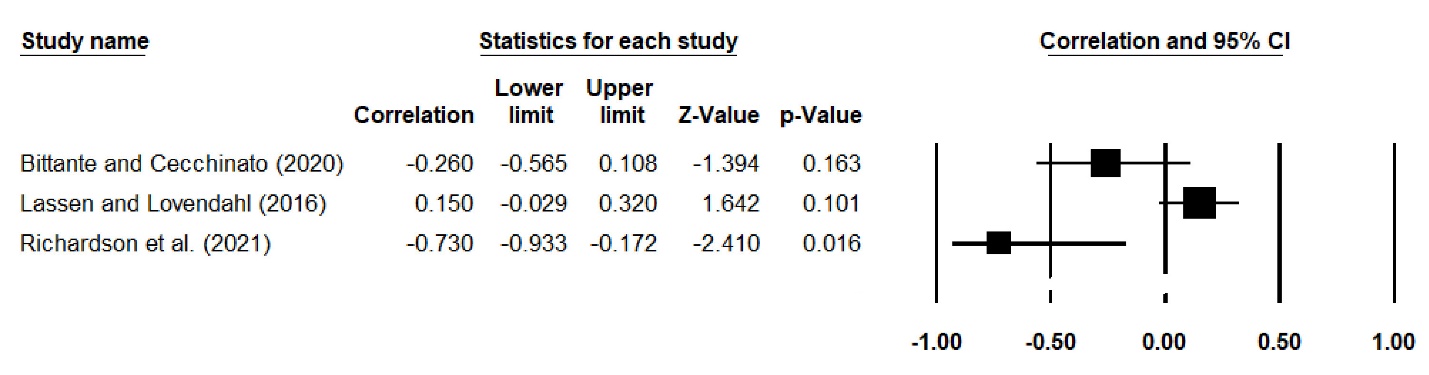


**Figure S3.** The forest plot of individual studies for genetic correlation estimates between METINT-CMY in dairy cows. Details are provided in Figure 2.


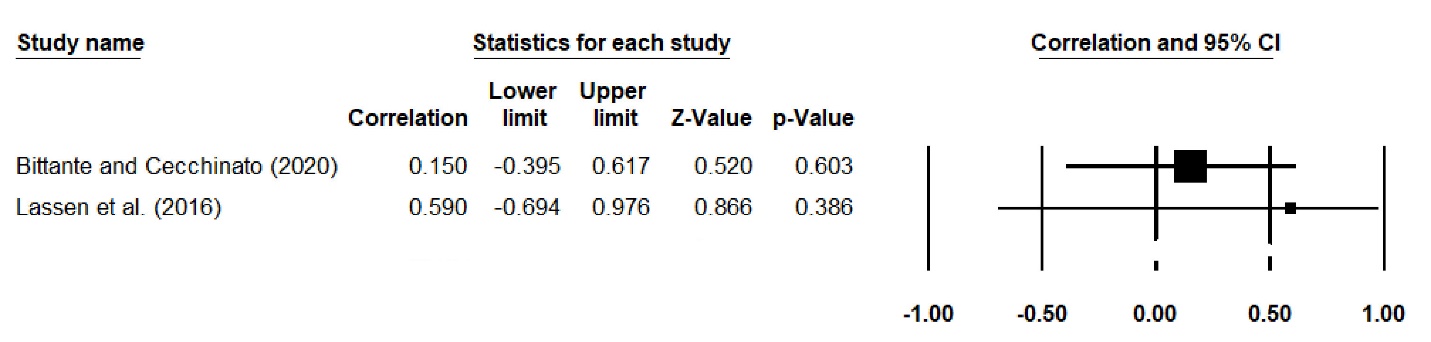


**Figure S4.** The forest plot of individual studies for genetic correlation estimates between METINT-Fatp in dairy cows. Details are provided in Figure 2.


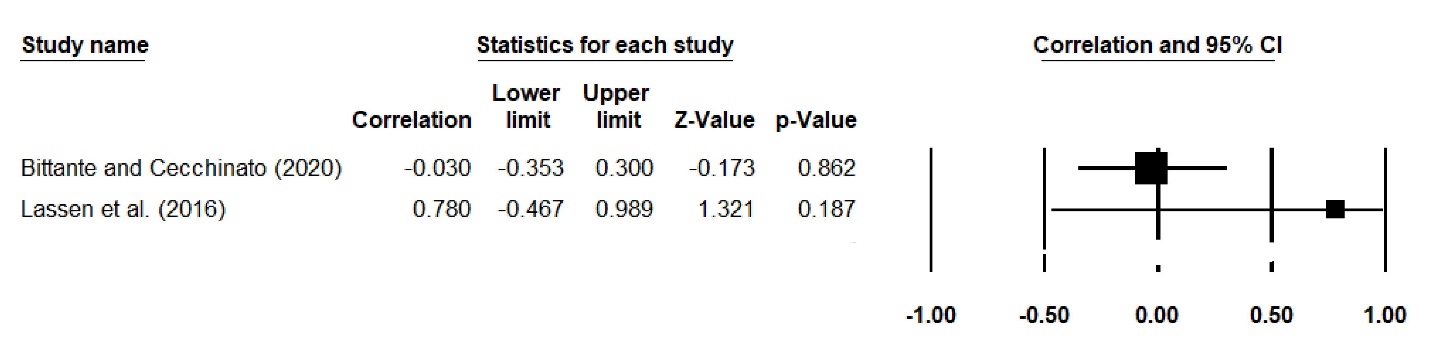


**Figure S5.** The forest plot of individual studies for genetic correlation estimates between METINT-Prop in dairy cows. Details are provided in Figure 2.


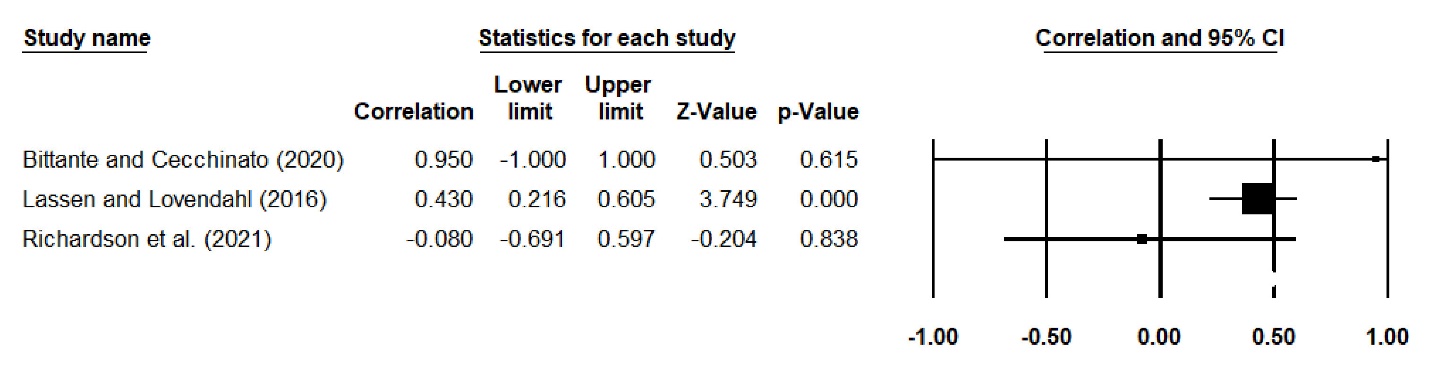


**Figure S6.** The forest plot of individual studies for genetic correlation estimates between METP-CMY in dairy cows. Details are provided in Figure 2.


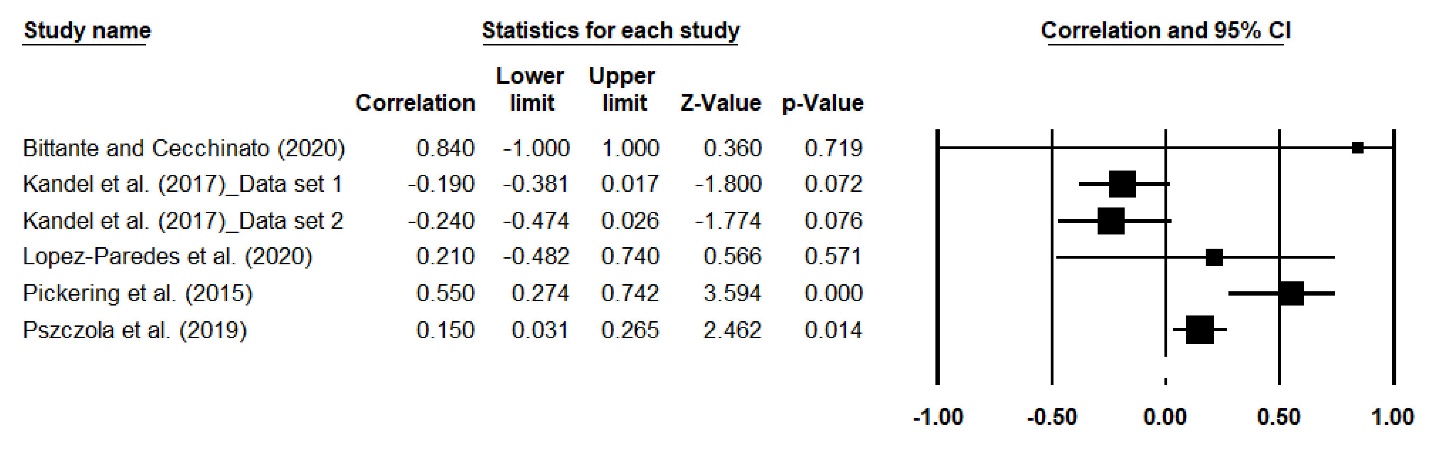


**Figure S7.** The forest plot of individual studies for genetic correlation estimates between METP-DMY in dairy cows. Details are provided in Figure 2.


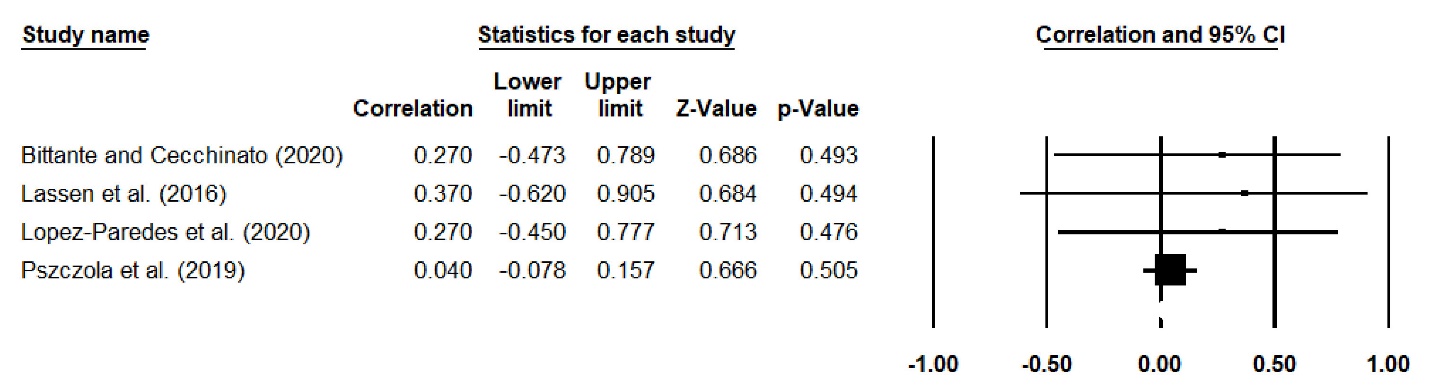


**Figure S8.** The forest plot of individual studies for genetic correlation estimates between METP-Fatp in dairy cows. Details are provided in Figure 2.


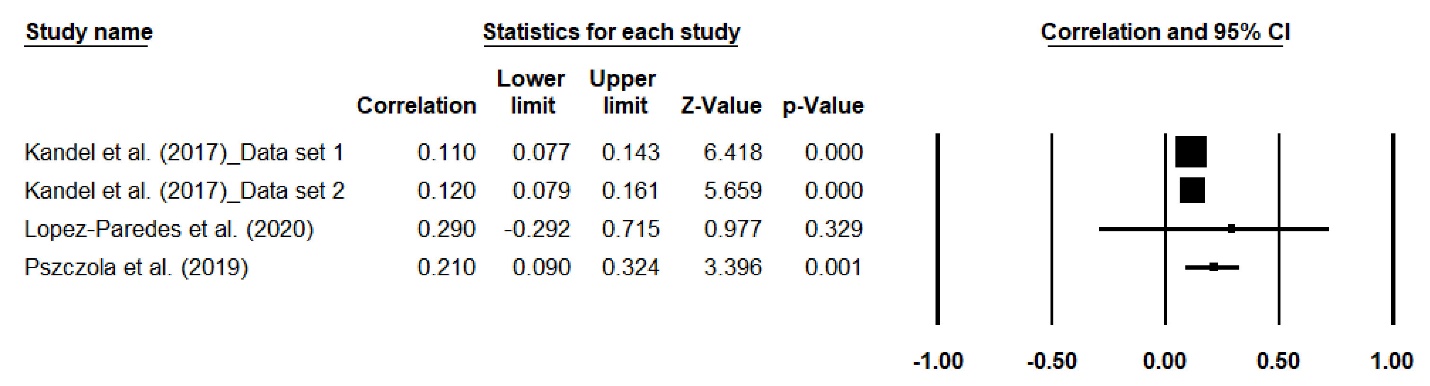


**Figure S9.** The forest plot of individual studies for genetic correlation estimates between METP-Faty in dairy cows. Details are provided in Figure 2.


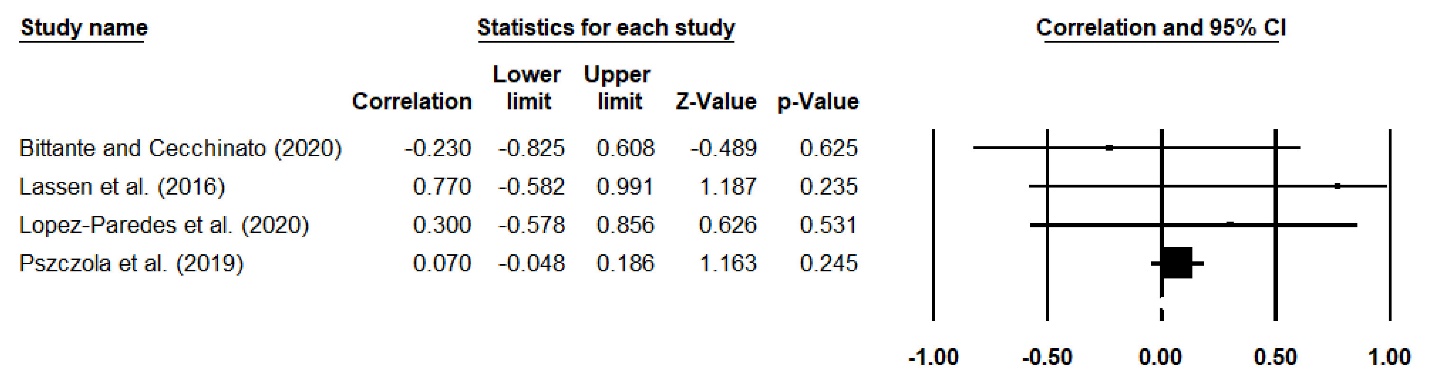


**Figure S10.** The forest plot of individual studies for genetic correlation estimates between METP-Prop in dairy cows. Details are provided in Figure 2.


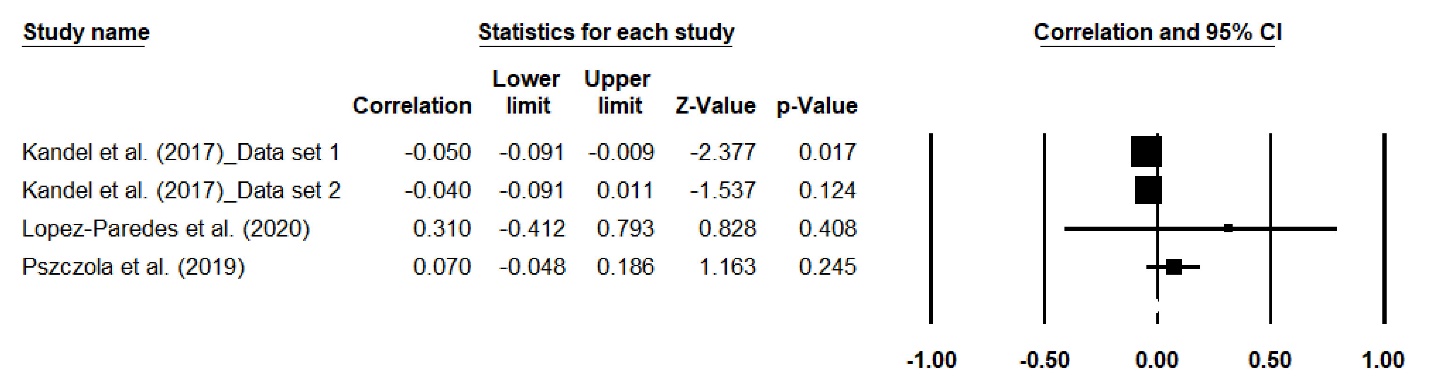


**Figure S11.** The forest plot of individual studies for genetic correlation estimates between METP-Proy in dairy cows. Details are provided in Figure 2.


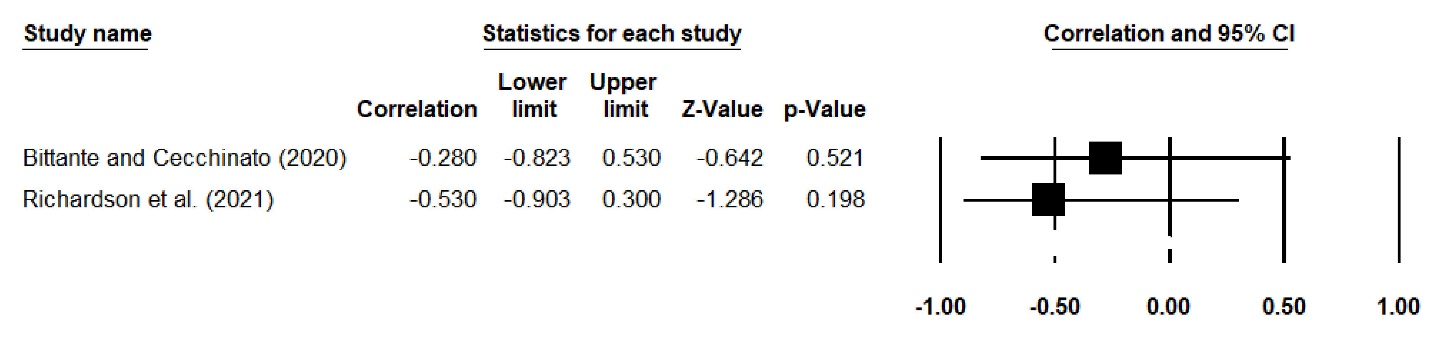


**Figure S12.** The forest plot of individual studies for genetic correlation estimates between METY-CMY in dairy cows. Details are provided in Figure 2.
